# An Autopsy Case With Fragile X‐Associated Tremor/Ataxia Syndrome Presenting Intranuclear Inclusion Bodies Mainly in the Limbic System

**DOI:** 10.1111/neup.70053

**Published:** 2026-03-16

**Authors:** Ayako Shioya, Kazuhiro Ishii, Taiki Sato, Masayuki Noguchi, Akira Tamaoka, Yuko Saito

**Affiliations:** ^1^ Department of Neurology, Division of Clinical Medicine, Faculty of Medicine University of Tsukuba Ibaraki Japan; ^2^ Department of Pathology, Faculty of Medicine University of Tsukuba Ibaraki Japan; ^3^ Department of Neurology Tsukuba Memorial Hospital Ibaraki Japan; ^4^ Department of Neuropathology (Brain Bank for Aging Research) Tokyo Metropolitan Institute for Geriatrics and Gerontology Tokyo Japan

**Keywords:** electron microscope, fragile X‐associated tremor/ataxia syndrome (FXTAS), Intranuclear inclusion, middle cerebellar peduncle (MCP) sign

## Abstract

Fragile X‐associated tremor/ataxia syndrome (FXTAS) is a neurodegenerative disorder characterized by a late onset and slow progression caused by a premutation (55–200 CGG repeat) in the fragile X mental retardation (*FMR1*) gene. Here, we report the case of a Japanese patient with FXTAS which is the first case autopsied in Japan. The patient was a 74‐year‐old man with a family history of fragile X syndrome at the time of death. The clinical features included postural tremors, mild cognitive decline, and ataxia. Magnetic resonance imaging (MRI) showed a high‐intensity lesion in the bilateral middle cerebellar peduncles and deep white matter around the ventricle on T2‐weighted images. A gene analysis revealed that the patient had a pre‐mutation of the CGG expansion (83 CGG repeats) in the *FMR1* gene. Neuropathologically, ubiquitin‐ and p62‐positive intranuclear inclusions were widely present, especially in the hippocampus. The middle cerebellar peduncle (MCP), where the “MCP sign” was seen on MRI, showed marked spongiosis with accompanied demyelination and axon loss, and a similar pathology was seen in the cerebral and cerebellar white matter. In an electron microscopy study, intranuclear inclusions were found to consist of a non‐membrane‐bound filamentous material. The clinical, MRI, and neuropathological findings were similar to those of neuronal intranuclear inclusion disease. Awareness of the disease is gradually increasing, and the number of autopsy cases is likely to increase, contributing to the elucidation of the pathology and development of treatments.

**Abbreviations**
Aβ: amyloid‐β
CGG: cytosine–guanine–guanine
FXAND: fragile X‐associated neuropsychiatric disorders
FXPOI: fragile X‐associated primary ovarian insufficiency
FXS: fragile X syndrome
FXTAS: fragile X‐associated tremor/ataxia syndrome
FMR1: fragile X mental retardation 1
FMRP: fragile X mental retardation protein
GFAP: glial fibrillary acidic protein
MCP: middle cerebellar peduncle
MRI: magnetic resonance imaging
MSA: multiple system atrophy
NIID: neuronal intranuclear inclusion disease
p62: sequestosome 1
ROS: reactive oxygen species
RAN: repeat‐associated non‐AUG
SCA: spinocerebellar ataxia
SUMO: small ubiquitin‐related modifier
T2WI: T2‐weighted imaging

## 1 Introduction

Fragile X‐associated tremor/ataxia syndrome (FXTAS) is a neurodegenerative disorder characterized by a late onset and slow progression caused by a premutation (55–200 CGG repeat) in the fragile X mental retardation (*FMR1*) gene located on the X chromosome at Xq27.3 [1]. *FMR1* premutation also causes fragile X‐associated primary ovarian dysfunction (FXPOI) and fragile X‐associated neuropsychiatric disorders (FXAND) [2]. Premutation alleles translate into a two‐to‐eight‐fold increase in *FMR1* mRNA, inducing a toxic RNA gain of function [3]. In contrast, fragile X syndrome (FXS) is caused by a full mutation (> 200 CGG repeats) and subsequent epigenetic silencing of the *FMR1* gene. Since the gene is silent, most affected individuals experience intellectual disability, problematic behaviors, and psychiatric comorbidities [4].

FXTAS typically presents with progressive cerebellar ataxia, postural tremor, parkinsonism, and mental symptoms [5]. Magnetic resonance imaging (MRI) mostly demonstrates middle cerebellar peduncle (MCP) and cerebral periventricular white matter hyperintensities in T2‐weighted images (T2WI), and T2 high‐signal region of the MCP, called “MCP sign,” which is a characteristic finding in FXTAS [6].

Neuropathological features of FXTAS include intranuclear inclusions in neurons and astrocytes [7, 8]. Intranuclear inclusions are positive for ubiquitin staining, and the number of inclusions correlates with the number of CGG repeats [1, 9]. In the electron microscope, the morphology of the intranuclear inclusion is observed as a granulofilamentous material without a capsule, and the inner filament is formed of 12–15 μm straight filament (non‐helical) [7]. The distribution of intranuclear inclusions is widespread, including the cerebral cortex, brainstem, and cerebellum [7, 9]. Furthermore, cerebral subcortical and cerebellar white matter diseases are characteristic findings. White matter diseases are widespread in the cerebrum, cerebellum, MCP, and brain stem [7, 9].

In this report, we experienced the first autopsy case of FXTAS in Japan that showed postural tremors, cognitive decline, and ataxia, and found many inclusions in neurons and astrocytes of the hippocampus.

## 2 Clinical Summary

A 74‐year‐old man died in a hospital. His brother also had Parkinson's disease. His grandchild had FXS and was diagnosed with attention‐deficit/hyperactivity disorder.

At the age of 63 years, right‐hand postural tremors began, and he was diagnosed with essential tremors at a clinic, which improved with the administration of arotinolol hydrochloride. However, the tremor slowly progressed, and the left arm postural tremor started at the age of 68 years. At 71 years of age, neurological findings showed a mild cognitive decline, truncal‐dominant ataxia, ataxic gait, and ataxic speech. He also had postural and action tremors. Muscle tonus and tendon reflexes in the extremities were normal and there were no muscle weakness, sensory disturbance, or autonomic dysfunction. Brain MRI showed a high‐intensity lesion in the bilateral MCPs and deep white matter around the ventricle on T2WI and fluid‐attenuated inversion recovery image (FLAIR). Diffusion‐weighted imaging showed no high‐intensity signal at the corticomedullary junction. In addition, atrophy was identified in the cerebral cortex, vermis, and cerebellar hemisphere. Genetic analysis of *FMR1* revealed 83 CGG repeats (normal and intermediate < 55 repeats) showing abnormal expansion. Thus, the patient was diagnosed with FXTAS. A detailed clinical history of up to 71 years has been reported [6]. In MRI performed approximately 3 years later, atrophy of both cerebral hemispheres and cerebellar hemispheres has progressed. Furthermore, deep white matter lesions observed on T2‐weighted images have diffusely expanded. Subsequently, pancreatic cancer was discovered during follow‐up at an outpatient clinic, resulting in death at 74 years. The total clinical course of the FXTAS was 11 years.

## 3 Pathological Findings

An autopsy was performed 2 h and 12 min after death with the consent of the bereaved family. The primary pathology of the general autopsy revealed pancreatic cancer as the cause of death.

Neuropathologically, the right side of the brain was frozen for biochemical and molecular biological studies, whereas the left side was fixed in 20% formalin for 1 month for examination. Coronal sections of the cerebrum, sagittal sections of the cerebellum, and vertical sections of the brainstem and spinal cord were performed. After fixation, brains were sliced into representative areas and embedded in paraffin. Slices were cut at a thickness of 6 μm with hematoxylin and eosin and Klüver Barrera stain.

Immunostaining was performed using an automated immunostainer (Ventana XT DISCOVERY; Ventana, Tucson, USA). Antibodies used were against ubiquitin (Z0485, rabbit monoclonal, Dako, Glostrup, Denmark), p62 (SQSTM1, rabbit polyclonal, MBI, Nagoya, Japan), human macrophage (CD68, mouse monoclonal, Dako, Glostrup, Denmark), phosphorylated neurofilament (SMI31, mouse monoclonal, Sternberger Immunochemicals, Bathimore, MA, USA), glial fibrillary acidic protein (GFAP, rabbit monoclonal, Dako, Glostrup, Denmark), phosphorylated α‐synuclein (pSyn#64, mouse monoclonal, FUJIFILM Wako Pure Chemical Corporation, Osaka, Japan), phosphorylated tau (ptau; AT8, mouse monoclonal, Innogenetics, Gent, Belgium), phosphorylated TDP‐43 (pSer409/410, mouse monoclonal; a gift from M. Hasegawa, Japan) [10], and amyloid‐β (Aβ) (12B2, mouse monoclonal, IBL, Maebashi, Japan). Neuropathological examinations were performed according to the Brain Bank for Aging Research [10]. In addition, pieces of formalin‐fixed brain tissue from the hippocampus and adrenal gland were fixed with glutaraldehyde and embedded in the epon for electron microscopy.

**FIGURE 1:**
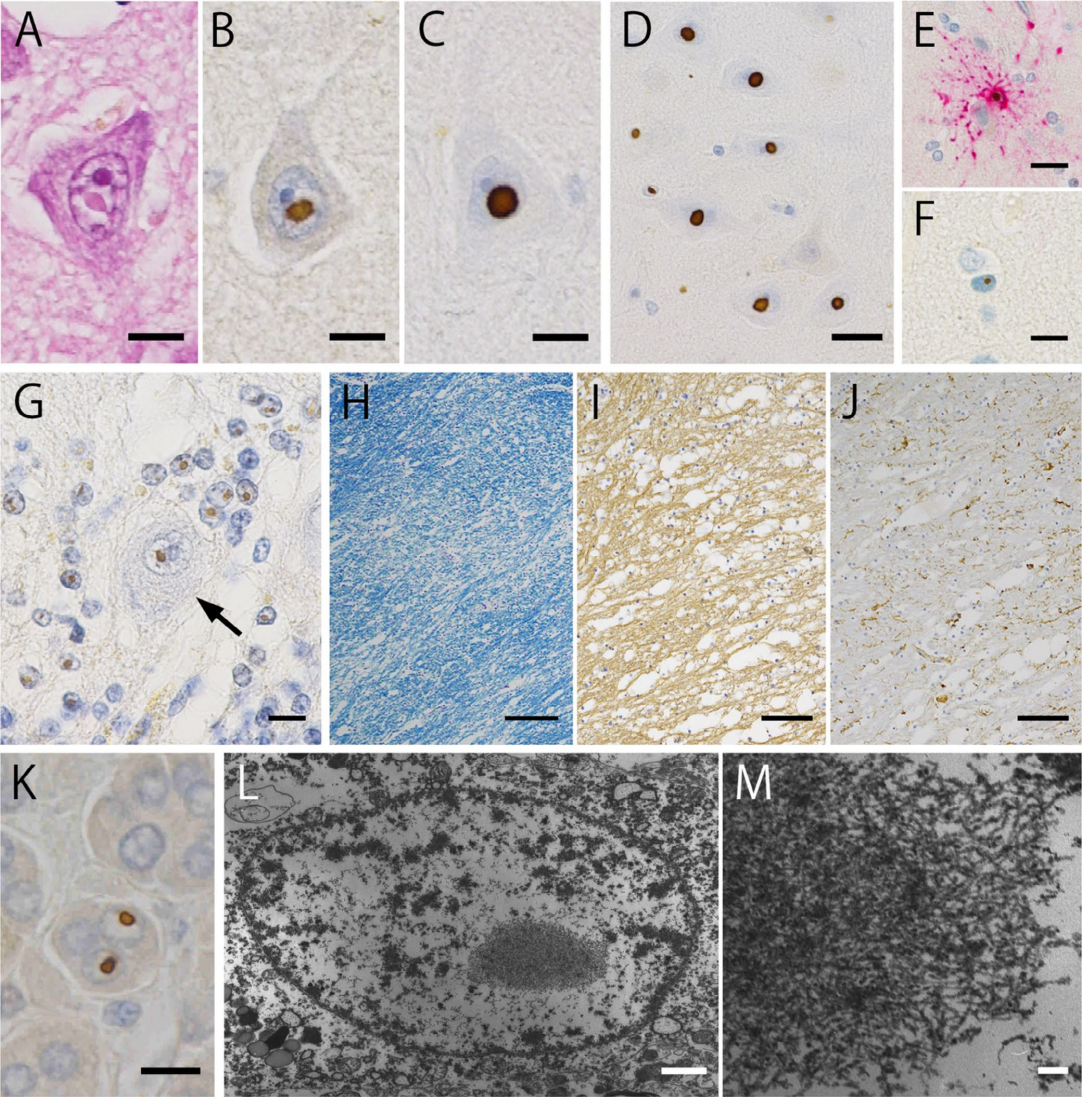
Histologic findings. (A–C) From 2 to 5 μm in diameter, circular to oval, eosinophilic inclusion bodies are observed in Hematoxylin–eosin stain (A). The cells are immunopositive for anti‐ubiquitin (B) and p62 antibody (C) antibodies. (Bar = 10 μm). D. The inclusions in CA4 of the hippocampus. Abundant Intranuclear Inclusion Bodies in Neurons. (anti‐p62 immunostaining, bar = 20 μm). E, F. Intranuclear inclusions in the astrocyte (I) and oligodendroglia (J) (red; anti‐GFAP and brown; anti‐p62 double‐immunostaining. Upper inset: Bar = 20 μm, Lower inset: Bar = 10 μm). G. Purkinje cell layers in the cerebellar hemisphere. Activation of Bergmann glia is also observed. Many neuronal intranuclear inclusions are observed, which are also scattered in the Purkinje cells (arrow). (anti‐ubiquitin immunostaining) bar = 10 μm. (H–J) Decreased myelin staining is polar in the MCP (H), and axon loss is observed (I). However, the activation of astrocytes is not mild compared to these changes (J) (F: Klüver‐Barrera staining. Bar = 200 μm, G: Anti‐SMI‐31 immunostaining，bar = 100 μm, H: Anti‐GFAP immunostaining，bar = 100 μm). K. Intranuclear inclusions in the adrenal cell are seen. They are ubiquitin‐positive according to immunohistochemistry analysis. (bar = 10 μm). (L, M) Electron microscopic studies from the inclusion of the astrocyte in the hippocampus at lower magnification (L) and higher magnification (M). The inclusions are fibrillary, granular, and not membrane‐bound. (L:Bar = 1 μm, M:Bar = 50 nm). (N, O) Electron microscopic images of the adrenal gland at lower (N) and higher magnifications (O). No capsules in the inclusions or beaded fibers are arranged randomly. (N:Bar = 1 μm, O:Bar = 50 nm).

A gross examination of the brain, which weighed 1170 g before fixation, revealed atrophy in the cerebrum, including the precentral gyrus, cerebellum, and brainstem. Furthermore, the corpus callosum was thin and the lateral and fourth ventricles were dilated. Microscopic examination revealed eosinophilic intranuclear inclusions in the cerebrum positive for ubiquitin and p62 (Figure 1A–D). Similar inclusions were observed in both neurons (Figure 1A–D), astrocytes (Figure 1E), and oligodendroglia (Figure 1F). In the cerebellum, the loss of Purkinje cells was accompanied by an empty basket, torpedo, and Bergmann gliosis. In the Purkinje cell layer, intranuclear inclusions were prominently observed in the Bergmann glia and remaining Purkinje cells (Figure 1G, arrow). The MCP, where the “MCP sign” was seen on MRI, showed marked spongiosis with accompanied demyelination and axon loss (Figure 1H,I). In addition, GFAP‐positive reactive astrocytes decreased (Figure 1J) and CD68‐positive microglia/macrophages were also sparse compared to tissue changes. Although similar pathology was observed in the cerebral and cerebellar white matter, the changes were milder than those observed in the MCP. Senile plaques were seen in the occipital and parietal cortex (Aβ), and neurofibrillary tangles were only scattered in the transentorhinal cortex (AT8). No positive inclusions were observed (TDP‐43), and Lewy bodies were absent (pSyn#64). We counted the neurons and astrocytes with intranuclear inclusions at a 400× field of view to investigate the distribution of intranuclear inclusions in the central nervous system. The content rate was calculated after 15 places (Table 1).

**TABLE 1:** Counts of intranuclear inclusions in neurons and astrocytes.

Brain area	Neurons			Astrocytes		
	Cell count with inclusion body	Total cell count	(%)	Cell count with inclusion body	Total cell count	(%)
Frontal cortex	15	157	9.6	53	536	9.9
Temporal cortex	10	186	5.4	72	534	13.5
Calcarine cortex	8	129	6.2	46	741	6.2
Caudate nucleus	18	226	8.0	29	294	9.9
Putamen	2	106	1.9	8	262	3.1
Globus pallidus	0	17	0	10	468	2.1
Amygdala	10	137	7.3	37	343	10.8
Hippocampus	64	224	28.6	100	310	32.3
Dentate gyrus	162	1527	10.6	—	—	—
Basis pontis	1	111	0.9	22	661	3.3
Substantia nigra	10	64	15.6	7	275	2.5
Dentate nucleus	7	148	4.7	14	601	2.2
Purkinje cell layer	5	64	7.8	342	972	35.2

In the visceral organs, intranuclear inclusions similar to those observed in the central nervous system were observed in the testis and adrenal glands (Figure 1K), kidney, pancreas, urinary tract, myocardium, coronary arteries, gall bladder, bladder, prostate, stomach, and small intestine, but not in the lungs, liver, and spleen (skin was not sampled).

Electron microscopy revealed that the intranuclear inclusion in the hippocampus consisted of non‐membrane‐bound filamentous material (Figure 1L,M).

### 3.1 Ethics Statement

The analysis of this case was approved by the Ethics Committee of the National Center of Neurology and Psychiatry (approval number: 2014‐508). Written informed consent for autopsy and research use was obtained from the next of kin.

## 4 Discussion

Here, we describe the postmortem features of a genetically confirmed carrier of *FMR1* premutation. The patient clinically presented with postural tremors, mild cognitive decline, and ataxia, and showed typical MRI findings (signal change in the cerebral white matter and MCP). These findings met the diagnostic criteria (two major clinical criteria [intention tremor and cerebellar gait ataxia] and one major radiological criterion [MRI white matter lesions involving the MCP]) for FXTAS [11]. Microscopically, typical ubiquitin‐ and p62‐positive intranuclear inclusions were spread extensively, especially in the hippocampus, and white matter changes were observed in the cerebrum and cerebellar white matter, including the MCP.

The neuropathological hallmark of FXTAS is the presence of intranuclear inclusions, which usually contain *FMR1* mRNA, polyG peptides, lamins A and C, ubiquitin, small ubiquitin‐related modifier (SUMO), and p62 proteins, whereas it is rare to find polyglutamine (polyQ) and negative for FMRP or TDP43 [12]. Several neuropathological studies have demonstrated the distribution of nuclear inclusions in neurons and astrocytes [7, 8, 9]. Greco counted the intranuclear inclusions in various brain regions in two FXTAS autopsy cases and calculated the percentage of neurons with nuclear inclusions from the total cell count [7]. The results showed that more abundant inclusions were present in the hippocampus (Case 1, 37.6%; Case 2, 43.0%) and secondary substantia nigra (Case 1 only; 10.5%). Louis also described the proportion of intranuclear inclusions in neurons [8] and found that intranuclear inclusions were more abundant in the Brodmann area 9 (20.9%), Brodmann area 28 (24.4%), hippocampus (23.8%), amygdala (22.8%), and inferior olivary nucleus (35.2%). In our case, neuronal intranuclear inclusions were more abundant in the hippocampus (28.6%), substantia nigra (15.6%), and dentate gyrus (10.6%); however, they were less abundant in the amygdala (7.3%) and were not counted in the inferior olivary nucleus.

In Japan, the prevalence of FXTAS is yet to be clarified because FXTAS may remain underdiagnosed given its rarity, lack of recognition, and overlap of symptoms with other neurodegenerative disorders, such as spinocerebellar ataxia, multiple system atrophy (MSA), and progressive supranuclear palsy [2]. In a previous study, the prevalence of FXTAS was 0.3% among those with ataxia after excluding patients who were negative for the most common Spinocerebellar Ataxia (SCA) subtypes and a Pro102Leu mutation in the PRNP gene responsible for the Gerstmann‐Sträussler‐Scheinker syndrome (GSS‐P102L), which was lower than that in other countries [2]. Although one case was reported in which a skin biopsy had been performed [13], autopsy cases have only been performed in other countries, and this case was the first report in Japan.

In recent years, the etiology of FXTAS has gradually become clear, and two major mechanisms have been proposed. First, the toxicity of elevated *FMR1* mRNA, which has several mechanisms, including the sequestration of proteins that are important for neuronal function, dysregulation of intracellular calcium levels, and degradation of mitochondrial function, leading to the production of reactive oxygen species (ROS) and oxidative stress [4, 14, 15]. Second, neurodegeneration caused by repeat‐associated non‐AUG initiated (RAN) translation and the production of a protein produced by RAN translation, which generates an out‐of‐frame peptide that contains a polyglycine tail called FMRpolyG, is toxic to brain cells [16, 17].

Neuronal intranuclear inclusion disease (NIID) is a slowly progressive neurodegenerative disease that is caused by non‐cording CGG trinucleotide repeat expansions in the 5′ untranslated region of NOTCH2NLC (Notch 2N‐terminal like C) [18, 19]. FXTAS and NIID are similar with overlapping clinical features and MRI findings [20]. Furthermore, the pathological changes are similar, and eosinophilic ubiquitin‐positive intranuclear inclusions are present in the central nervous system and visceral organs in both diseases. Consequently, these disorders were hypothesized to share a similar molecular base caused by non‐cording trinucleotide repeat expansions. Previously, it was difficult to distinguish between the two diseases using only histopathological findings, and genetic analysis of the *FMR1* gene was the only means to distinguish NIID from FXTAS [20]. However, the identification of the pathogenic gene of NIID, NOTCH2NLC, facilitated the differentiation of both pathologies. In our case, the clinical presentation, MRI, and neuropathological findings were like those of NIID, and only the family history was a clue to suspect FXTAS.

In visceral organs of FXTAS including our case, intranuclear inclusions had been reported in the adrenal glands, testis, epididymis, ganglion cells (dorsal root ganglia, paravertebral sympathetic ganglia, gastric myenteric ganglia, epicardial autonomic ganglia), heart, kidney, pancreas, thyroid, prostate, bladder, gall bladder, and adipose cell [21, 22, 23]. In addition to these organs, the intranuclear inclusions were seen in the liver, lung, and spleen in NIID, and intranuclear inclusions were seen in more organs than seen in FXTAS. Although the clinical‐pathological correlation in visceral organs had not been established [23], it might be necessary to examine the relationship between clinical symptoms and distribution of the intranuclear inclusions.

Neuronal intranuclear inclusion disease (NIID) is currently recognized as a highly heterogeneous disorder, exhibiting substantial variability in genetic background, clinical phenotype, and pathological involvement. Although GGC repeat expansion in NOTCH2NLC has been identified as a major genetic cause in many cases, previous studies have demonstrated marked heterogeneity in repeat length, sequence characteristics, and phenotypic expression, resulting in diverse clinical manifestations and imaging findings across patients. Such observations suggest that NIID represents a broad clinicopathological spectrum rather than a single uniform disease entity [24, 25].

Recent comprehensive reviews of NOTCH2NLC‐related repeat expansion disorders have further emphasized the wide phenotypic and pathological spectrum associated with NIID, including variability in neurological involvement, systemic organ distribution, and disease severity, reinforcing the concept of NIID as an intrinsically heterogeneous condition [26].

In conclusion, we described the neuropathological findings in a patient with FXTAS who was first autopsied in Japan. Ubiquitin‐ and p62‐positive intranuclear inclusions were broadly present, especially in the hippocampus. The MCP, where the “MCP sign” was seen in MRI, showed marked spongiosis with accompanied demyelination and axon loss, and a similar pathology was seen in the cerebral and cerebellar white matter. Recognition of the disease is gradually increasing and the number of autopsy cases is likely to increase in the future, which will contribute to elucidating its pathology and developing treatments.

## Funding

This work was supported by JSPS KAKENHI (Grant Number JP22H04923 [CoBiA]); the Integrated Research Initiative for Living Well with Dementia (IRIDE) of the Tokyo Metropolitan Institute for Geriatrics and Gerontology; the Japan Agency for Medical Research and Development (AMED) under Grant Numbers JP24wm0425019 and JP24dk0207074h0001; and the MHLW Research on Rare and Intractable Diseases Program (Grant Number JPMH23FC1008). Grant JP24dk0207074h0001 also provided support to M.H.

## Conflicts of Interest

The authors declare no conflicts of interest.

## Data Availability

The data that support the findings of this study are available from the corresponding author upon reasonable request.
